# Thyrotoxicosis-Induced Cardiogenic Shock: Acute Management Using a Multidisciplinary Approach

**DOI:** 10.7759/cureus.32841

**Published:** 2022-12-22

**Authors:** Oluwaremilekun Z Tolu-Akinnawo, Joseph Abiade, Tiwalade Awosanya, Henry E Okafor

**Affiliations:** 1 Internal Medicine, Meharry Medical College, Nashville, USA; 2 Endocrinology, Meharry Medical College, Nashville, USA; 3 Cardiology, Vanderbilt University Medical Center, Nashville, USA

**Keywords:** acute management of thyrotoxicosis, multi-disciplinary team approach, role of beta-blockers, cardiogenic shock, management of thyrotoxicosis

## Abstract

The development of heart failure and cardiomyopathy has been identified as an infrequent but life-threatening complication of thyrotoxicosis or thyroid storm. Thyrotoxicosis-induced cardiomyopathy and cardiogenic shock have been shown to be one of the major causes of sudden mortality in adults. However, the treatment of thyrotoxicosis with non-cardioselective beta-blockers has been implicated in the development of severe decompensation and even cardiogenic shock if cardiac function is not known and often requires a multidisciplinary care team to address it.

Here, we have reported the case of a 63-year-old male with a past medical history of hyperthyroidism who presented to the emergency room with persistent shortness of breath. Vital signs were notable for hypotension, tachycardia with an irregular heartbeat, with ECG suggestive of atrial fibrillation with a rapid ventricular rate. The thyroid function test was significant for severely suppressed TSH, and the Burch-Wartofsky Score was >45. The patient rapidly decompensated shortly after being given IV metoprolol, subsequently requiring intubation and pressor support. Two-dimensional (2D) echocardiography (or echo) done afterward was significant for four-chamber dilation with mild global hypokinesis and reduced left ventricular ejection fraction. Endocrinology, Cardiology, and Pulmonary Critical Care teams were consulted to assist in multi-modality management. The administration of a non-cardioselective beta-blocker in decompensated heart failure was suggested as the cause of the rapid deterioration. Through a multi-modality management approach, the patient subsequently improved and was eventually discharged with the resolution of thyroid storm and cardiogenic shock, and with close follow-up with the primary care provider, endocrinologist, and cardiologist. This case illustrates the significance of a multidisciplinary team approach in the acute management of thyrotoxicosis-induced cardiogenic shock, as recommendations from the team were instrumental in helping the patient recover from the acute phase of the illness. Also, this case further highlights the significance of assessing the cardiac function, preferably performing echo before starting the patient on beta-blockers.

## Introduction

Heart failure is a rare but grave complication of thyrotoxicosis or thyroid storm. About 6% of patients with thyrotoxicosis develop heart failure [1]. However, thyrotoxicosis-induced cardiogenic shock has a mortality rate as high as 30% [2]. The use of non-cardioselective beta-blockage in the treatment of thyrotoxicosis has been implicated in the development of severe cardiopulmonary decompensation and even cardiogenic shock if the cardiac function was not known prior to initiation [3]. Cardiogenic shock is an infrequent complication of thyrotoxicosis [3].

Here, we report a case that demonstrates that early detection and treatment align with the potential for nearly complete recovery if the patient survives the critical phase.

## Case presentation

This is a case of a 63-year-old male with a past medical history of chronic obstructive pulmonary disease (COPD), type 2 diabetes, and hyperthyroidism, non-compliant on home methimazole, who presented to the emergency room (ER) with a history of persistent shortness of breath; vital signs were notable for hypotension with blood pressure at 81/59 mmHg, tachycardia at 200 beats per minute, irregular, respiratory rate of 22 breaths per minute, and requiring 5 L of nasal cannula oxygen supplementation to keep saturation at >94%. On physical examination, he was found to be emaciated with decreased air entry in the right mid and lower lung zones, bilateral 3+ pitting pedal edema up to the thighs, and cold extremities.

**Figure 1:**
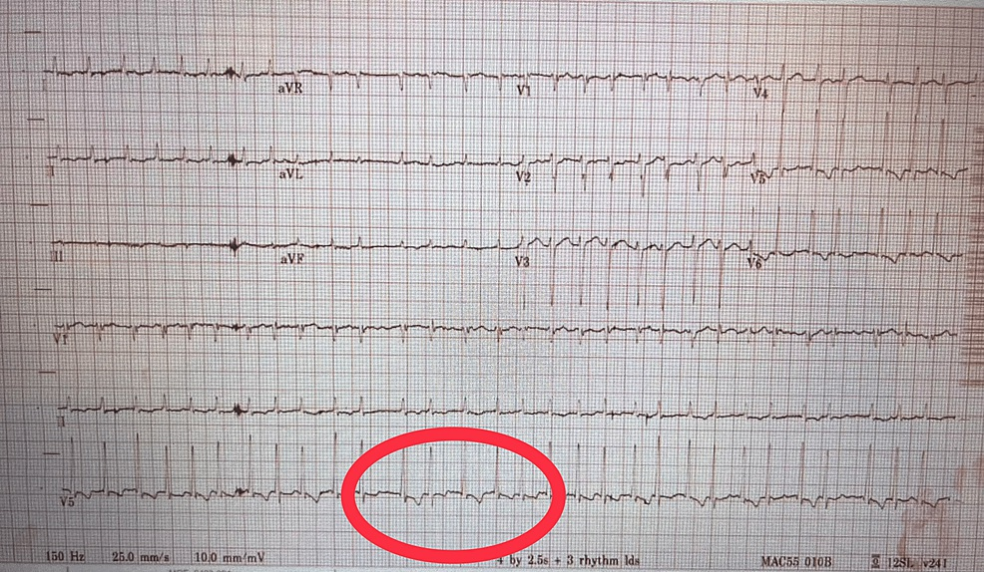
ECG revealing atrial fibrillation with a rapid ventricular rate (red circle) in the 180s

ECG revealed atrial fibrillation with a rapid ventricular rate in the 180s (Figure 1).

**Figure 2:**
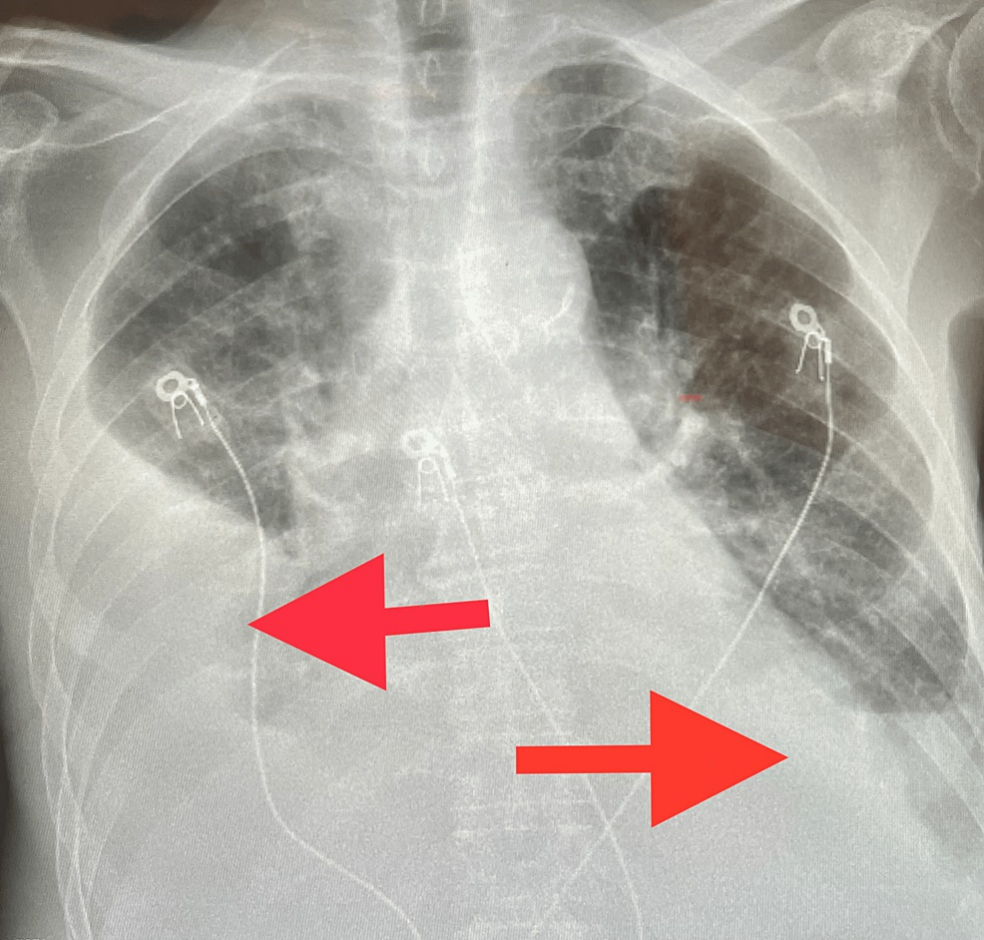
Chest X-ray showing large right-sided and moderate left-sided pleural effusion (red arrows), with bibasilar consolidation

Chest X-ray revealed large right-sided and moderate left-sided pleural effusion, with bibasilar consolidation and bilateral airspace disease (Figure 2).

Lab values were significant for anemia with a hemoglobin/hematocrit ratio of 11.6/34.7, mean corpuscular volume 80.9 fL, elevated alkaline phosphatase/aminotransferase level 145/52 U/L, elevated D-dimer level at 1.99 mg/L, hypomagnesemia with serum magnesium level 1.6 mg/dL, troponin level at 0.034 ng/mL, and brain natriuretic peptide level elevated at 5619 pg/mL. The thyroid function test revealed a severely suppressed thyroid stimulating hormone (TSH) level at <0.007 uU/mL with an elevated free thyroxine at 4.98 ng/dL. The Burch-Wartofsky Score was >45, which is quite sensitive but not very specific to diagnosing thyroid storm.

Of note, two weeks before admission, the patient had presented to an outside facility with shortness of breath. Two-dimensional (2D) echocardiography (or echo) revealed a left atrial appendage thrombus. The TSH level was reportedly severely depressed. He was started on acute management; however, the patient reportedly left against medical advice.

**Figure 3:**
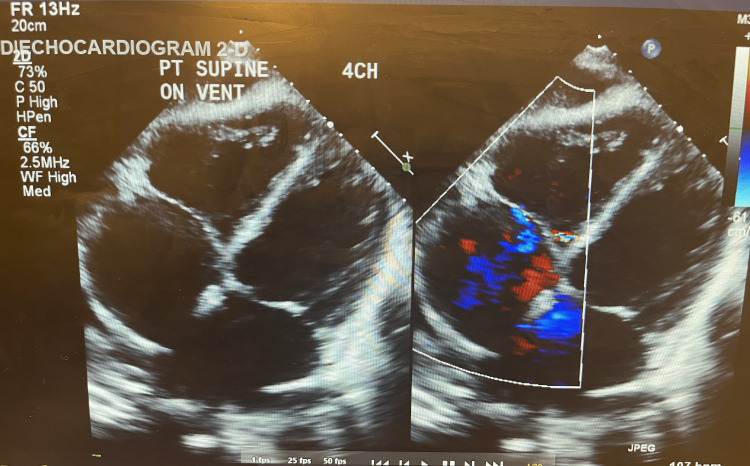
Two-dimensional echo significant for four-chamber dilation with mild global hypokinesis and a reduced LVEF of 40%-45% echo, echocardiography; LVEF, left ventricular ejection fraction

In the present case, the patient was admitted with a diagnosis of severe thyrotoxicosis versus thyroid storm. The patient was started on IV metoprolol in the ER and acutely decompensated. Due to the complexity of the case, a multi-modality approach was used, with pulmonology, cardiology, and endocrinology teams consulted. Right thoracentesis was performed, draining ~780 cc of straw-colored fluid. The patient, however, continued to have worsening shock/altered mentation and was subsequently intubated. 2D echo done at the bedside was significant for four-chamber dilation with mild global hypokinesis and a reduced left ventricular ejection fraction of 40%-45% (Figure 3).

As per cardiology recommendations, beta-blockers were held, and the patient was started on IV digoxin for rate control in the event of underlying heart failure. Vasopressors and inotropes were also used to keep the mean arterial pressure higher than 65 mmHg, with close monitoring and repletion of electrolytes. A heparin drip was also started due to the intracardiac thrombus finding on 2D echo.

In accordance with endocrinology recommendations, the patient was started on Lugol’s iodine that was helpful in the rapid decrease of free T3. The patient was also started on IV steroids that had an effect on inhibiting the conversion of T4 to T3. Propylthiouracil (PTU) was also started as a thyroid blocker.

With interval improvements in the clinical status, the patient was titrated off pressors, and IV steroids. The patient was started on guideline-directed medical therapy for heart failure per cardiology recommendations with metoprolol succinate, lisinopril, empagliflozin, and spironolactone as blood pressure tolerated. The heparin drip was also switched to apixaban, and PTU was switched to methimazole. The Physical and Occupational Therapy team was also consulted and assisted with rehabilitation. A significant interval improvement in the clinical status continued to be noted, and the patient was deemed stable for discharge after 16 days of hospitalization with plans to closely follow up with the primary care provider, endocrinologist, and cardiologist as an outpatient.

## Discussion

Pathophysiology

Literature reviews have established the effect of thyrotoxicosis or thyroid storm in the eventual development of heart failure and cardiomyopathy [1]. The major effects of thyrotoxicosis on the heart are through the direct and indirect actions of T3. As a result of hyperthyroidism, heart rate and left ventricular contractility increase while there is a decrease in systemic vascular resistance. The reduction in vascular resistance leads to a decrease in renal perfusion, which in turn activates the renin-angiotensin-aldosterone pathway. This, in turn, leads to an increased preload and increased cardiac output [4]. The above-mentioned mechanisms lead to a reduction in the myocardial contractile reserve, putting the patient at risk of high-output heart failure, which has been found to be common with hyperthyroidism/thyrotoxicosis [4]. It is important to note that this progression can also be seen in patients with no prior cardiac dysfunction.

Of note, studies have also shown that about 10%-25% of patients with hyperthyroidism/thyrotoxicosis develop atrial fibrillation [4]. As seen in the literature review, one of the primary electrical abnormalities observed in patients with thyrotoxicosis/thyroid storm at the time of presentation is atrial fibrillation, with a rapid ventricular response, as seen in our patient. This also can worsen the low myocardial reserve leading to heart failure [1].

Management

This case highlights the significance of a multidisciplinary team approach in acutely managing thyrotoxicosis-induced cardiogenic shock. The recommendations and the intervention of the pulmonologist, cardiologist, endocrinologist, and hospitalist were helpful in patient's recovery from the acute phase of the illness. This case also further highlights the significance of assessing the cardiac function, preferably through echo before starting the patient on a non-cardioselective beta-blocker. Although the traditional treatment of thyrotoxicosis includes non-selective beta-blockers, their use may be destructive in a patient with severely reduced stroke volume due to their negative inotropic, and chronotropic effects, especially in situations where controlling the heart rate is fundamental to maintaining cardiac output as in the case of our patient [4]. This is in line with the existing literature, with a total of 11 cases of beta-blocker-induced cardiogenic shock in thyroid crisis reported shortly after the administration of a non-cardioselective beta-blocker, commonly propranolol [5-6].

Excess thyroid hormones cause systolic and diastolic dysfunction through direct toxic effects [4]. Several studies have shown that antithyroid treatment can reverse the cardiomyopathy effects of thyrotoxicosis [7-8]. The main goal of management is to restore a euthyroid state that will aid in restoring cardiac function [9]. Lugol's solution was helpful in the rapid decrease in free T3 levels, which is responsible for most of the effect on the myocardium. Lugol's iodine solution is helpful in rapidly decreasing thyroid hormone levels and reducing glandular blood flow. Glucocorticoids inhibit the conversion of T4 to T3, hence lowering the activity of T3. PTU inhibits the synthesis of thyroid hormones by interfering with thyroid peroxidase (TPO) and blocks the conversion of T4 to T3 stored in peripheral tissues. PTU also acts as a thyroid blocker to decrease the retention of radioactive iodine in the thyroid.

Although the cardiology team considered cardioversion, this was impossible due to the presence of a left atrial appendage thrombus. As we know, non-cardioselective beta-blockers can be useful in the management of heart failure-related hyperthyroidism; in this case, they were cautiously introduced as they occasionally exacerbate symptoms as seen in this case.

## Conclusions

Cardiogenic shock is an infrequent complication of thyrotoxicosis and is associated with high mortality. But as illustrated in this case, early detection and involvement of a multidisciplinary team are critical to patients' acute management and survival, with the potential for nearly complete recovery if the patient survives the critical phase. Also, worth noting is that patients presenting with thyrotoxicosis-induced shock may not be suitable candidates for standard management with non-cardioselective beta-blockers as this may worsen their condition. Assessing cardiac function prior to starting a beta-blocker is critical in preventing worsening clinical presentation, especially in patients with hemodynamic instability. This case also emphasizes the need for additional studies to formulate a therapeutic regimen to reduce the mortality rate associated with such cases.
